# Labor and Delivery Complications in Women With Renal Calculi: Increased Risk and Prolonged Hospital Stay

**DOI:** 10.7759/cureus.104492

**Published:** 2026-03-01

**Authors:** Gina Toma, Katelyn Healey, Avani Sharma, Yogita M Dintakurthi, Kelsey A Keel

**Affiliations:** 1 Medical School, William Carey University College of Osteopathic Medicine, Hattiesburg, USA; 2 Epidemiology and Public Health, William Carey University College of Osteopathic Medicine, Hattiesburg, USA

**Keywords:** labor and delivery complications, length of hospital stay (los), pregnancy surveillance, renal calculi, utilization of health care

## Abstract

Background: Renal calculi, or kidney stones, are a common, non-obstetric cause of hospitalization during pregnancy, yet their impact on labor and delivery outcomes at a national level within the United States remains unclear. This study aims to explore the connection between labor and delivery complications and prolonged hospitalizations in pregnant women diagnosed with kidney stones.

Methods: We conducted a national cross-sectional analysis study by analyzing data from the 2022 Healthcare Cost and Utilization Project National Inpatient Sample (HCUP-NIS). Pregnant patients were identified using delivery-related diagnostic-related group codes. Renal calculi were defined using International Classification of Diseases-10 (ICD-10) code N20.0 and trimester-specific codes O26.831-O26.833. Variables included primary expected payer, race, patient location, socioeconomic status, gestational age, emergency department (ED) service indicator, transfer out indicator, and age at admission. Weighted multivariable logistic regression was used to evaluate associations between renal calculi and labor delivery complications (LDCs).

Results: The analysis included 698,366 cases, representing 3,491,830 cases after data were weighed. Among pregnant patients with renal calculi, 84.7% experienced labor and delivery complications compared to 28.8% of those without renal calculi (p < 0.001). Additionally, pregnant women with renal calculi had significantly longer hospital stays (5.12 ± 7.5 days vs. 2.56 ± 2.2 days, p < 0.001). Insurance status was significantly associated with both renal calculi and LDCs. Patients insured by Medicare (adjusted odds ratio (aOR) 1.58) and Medicaid (aOR 1.14) had higher odds of complications compared to other insurance types. Increased urbanicity and lower median household income quartiles were also associated with increased odds of LDCs.

Discussion: Renal calculi during pregnancy are associated with elevated risks of maternal complications, including increased morbidity, preterm delivery, and prolonged hospitalization. Enhanced surveillance and tailored interventions for high-risk or otherwise vulnerable groups may reduce these adverse outcomes.

## Introduction

Renal calculi, or kidney stones, occur frequently during pregnancy, but little is known about their impact on labor and delivery outcomes [1]. Renal calculi, commonly referred to as kidney stones or nephrolithiasis, are solid crystalline concretions that form in the renal collecting system or urinary tract when supersaturation of urine with stone‐forming solutes (like calcium, oxalate, phosphate, uric acid, or cystine) overcomes inhibitory factors (like citrate or magnesium) and leads to nucleation, growth, aggregation, and retention of crystals [2]. The etiology is multifactorial and includes dehydration, hypercalciuria, hyperoxaluria, hyperuricosuria, hypocitraturia, urinary pH abnormalities, dietary factors (e.g., high salt, high protein, high oxalate), and rare inherited disorders such as cystinuria or primary hyperoxaluria [3].

Clinically, renal calculi may be asymptomatic when small and nonobstructive [4]. However, they may present acutely with flank or renal colic pain that radiates to the groin, gross or microscopic hematuria, and dysuria [4]. Diagnosis is based on a combination of clinical presentation and investigations: urinalysis (hematuria, crystals, signs of infection), blood biochemistry (renal function, calcium, uric acid, electrolytes), and imaging [5]. Non-contrast CT scan (NCCT) is the gold standard for symptomatic stones, while ultrasound may be used as an initial or adjunctive modality, especially to avoid radiation [4]. Stone composition analysis (e.g., infrared spectroscopy) and metabolic workup (24‑hour urine studies) help guide secondary prevention [6].

Management depends on stone size, location, symptoms, and associated complications (e.g., obstruction, infection). Small stones (<4-6 mm) are often managed conservatively with high fluid intake, analgesia, and medical expulsion therapy (e.g., α-blockers [4]). Larger stones, or those causing obstruction or infection, require interventional therapies, such as extracorporeal shock wave lithotripsy (SWL), ureteroscopy (URS, with laser lithotripsy), or percutaneous nephrolithotomy (PCNL or mini-PCNL), depending on the stone burden and anatomy [6]. Innovations in stone removal, such as spinner devices, to efficiently clear fragments in URS, are an area of active research [7]. Prevention of recurrence is critical and involves lifestyle and medical interventions, including ensuring high fluid intake to achieve a substantial urine output, dietary modification (e.g., moderate calcium intake, low sodium, reduced oxalate, limited animal protein), control of risk factors (e.g., hyperparathyroidism, hyperuricemia), and targeted pharmacotherapy (e.g., thiazide diuretics, potassium citrate, allopurinol) tailored to the stone type and metabolic abnormalities [3]. 

Renal calculi are common and recurrent, with a broad spectrum from silent stones to life-threatening obstructive infection; prompt recognition, appropriate imaging and metabolic evaluation, interventional treatment when needed, and aggressive preventive strategies are key to reducing morbidity and recurrence [8].

Renal calculi complicate pregnancies and are the most common non-obstructive cause of abdominal pain requiring hospitalization in pregnant patients [9,10]. Physiologic changes during pregnancy, such as progesterone-mediated smooth muscle relaxation and mechanical compression of the ureters, increase urinary stasis and predispose pregnant women to stone formation and symptomatic obstruction [9,10]. A 2021 systematic review and meta-analysis of 4.7 million pregnancies estimated the incidence of renal calculi in pregnancy at 0.49%, which is roughly one case for every 204 pregnancies [10]. Similarly, a large Mayo Clinic cohort study showed that the risk of a first symptomatic stone formation increases during pregnancy, with the risk of stone formation rising during the second and third trimesters, peaking in late pregnancy, then returning to baseline within one year postpartum [11].

Beyond frequency and timing, renal calculi in pregnancy are associated with adverse maternal and fetal outcomes [2]. Studies have demonstrated increased rates of preeclampsia, urinary tract infections, low birth weight, preterm labor, and cesarean delivery among affected women [1,12]. Pregnant patients with kidney stones often require hospitalization, and compared to nonpregnant women, they experience longer hospital stays and more complex management courses [12,13]. Management options for pregnant women with renal calculi are limited by fetal safety considerations. Initial care is typically conservative, but up to about one in four affected pregnancies may require surgical interventions [10,14]. Recent series and systematic reviews support the relative safety of uteroscopy and other interventions when performed in experienced centers [10,14]. Together, these findings underscore that renal calculi are not only a urologic concern but also a potential driver of obstetric complications and health care utilization, providing important context for evaluating their impact on labor and delivery outcomes and hospital resource use. 

Despite the known maternal risks and the potential for increased healthcare costs, few large-scale studies have evaluated how the presence of renal calculi during pregnancy affects mode of delivery, hospital resource utilization, or hospital length of stay. This gap in the literature underscores the need for population-level analyses to clarify the impact of urolithiasis on delivery outcomes, especially as the healthcare system continues to shift toward value-based reimbursement models. The aim of this study is to investigate the relationship between increased labor and delivery complications and longer hospital stays in pregnant women who are diagnosed with renal calculi. 

## Materials and methods

Data from the 2022 Healthcare Cost and Utilization Project National Inpatient Sample (HCUP-NIS) were analyzed [15]. Several patient- and community-level variables were included to characterize the population, including primary expected payer, race, patient location, socioeconomic status, gestational age, emergency department (ED) service indicator, transfer out indicator, and age at admission [15]. Primary expected payer was defined as the anticipated source of payment for the hospital stay, categorized as Medicare, Medicaid, private insurance, self-pay, no charge, or other [15]. In the HCUP-NIS, race is categorized as White, Black, Hispanic, Asian or Pacific Islander, Native American, and Other individuals [15]. The “Other” category includes individuals classified as multiracial, belonging to racial groups not explicitly listed in HCUP standard categories, or reported as “Other” by the originating hospital or state data source, as standardized by HCUP uniform coding. Patient location was classified using the National Center for Health Statistics (NCHS) urban-rural code, which distinguishes counties as large central metropolitan, large fringe metropolitan, medium metropolitan, small metropolitan, micropolitan, or noncore (rural) [15]. Socioeconomic status was measured using median household income quartiles for the patient’s ZIP Code, which is divided into four national quartiles, from lowest to highest, and derived from U.S. Census data. Pregnancy-specific variables included gestational age, recorded in completed weeks at admission or delivery, and categorized as preterm (<37 weeks), term (37-41 weeks), or post-term (≥42 weeks), and age in years at admission, as a continuous variable. The HCUP ED service indicator identified whether a patient received ED services during the admission, allowing distinction between patients admitted through the ED versus other sources of admission. The transfer out indicator specified whether the patient was discharged or transferred to a short-term hospital, critical access hospital, or other acute care facility. 

The outcome of interest was labor and delivery complications experienced in vaginal or cesarean births, as determined by Diagnostic-Related Group billing codes available in the patient record (International Classification of Diseases-10 (ICD-10)) [15]. Specifically, kidney stones were defined using N20.0 (calculus of the kidney) [15]. Trimester-specific codes were included to capture episodes during pregnancy: O26.831 (renal calculus complicating pregnancy, first trimester), O26.832 (second trimester), and O26.833 (third trimester). These codes encompass both general kidney stone diagnoses and those specifically linked to obstetric care during pregnancy. The independent variable was the presence of a diagnosis of kidney stones during pregnancy, identified using the ICD-10 diagnostic codes recorded in the patient’s discharge record. Patients with any of these ICD-10 codes listed as a principal or secondary diagnosis during the index hospitalization were categorized as having exposure of interest. 

We applied discharge-level weights to account for the complex survey design of the HCUP-NIS. All analyses used sampling weights provided in HCUP-NIS and were conducted using SAS v.9.4 software (SAS Institute, Cary, NC), with p-values < 0.05 considered statistically significant. Univariate statistics were calculated, and categorical variables were reported as frequency and percentage. Descriptive statistics for continuous variables were reported with mean and standard deviation (SD), or median and range as appropriate. Bivariate analyses were conducted to determine if each variable was independently associated with the outcome. Variables found to be significantly associated, with a p-value of <0.001, were included in a multivariable model. Backward conditional binary logistic regression was performed to step down the model until all variables remaining in the model were significant at p < 0.001. 

## Results

A total of 698,366 cases were included in the analysis, representing 3,491,830 cases after data were weighted. Nearly half of the study population (n = 1,697,590, 48.6%) were young adults between 21 and 30 years of age, representing the largest age group in the cohort. The majority of pregnant women were White women (n= 1,722,425, 49.3%), carried private health insurance (n= 1,841,340, 52.7%), and most resided in communities within the lowest national quartile of median household income ($1-$57,488; 26.8%).

Overall, 6,510 (84.7%) mothers with renal calculi experienced labor delivery complications (LDCs), whereas 1,003,060 (28.8%) mothers without renal calculi experienced LDCs (p < 0.001), as indicated in Table 1. Among those with LDCs, approximately one-third had an ED revenue code documented, and roughly one-quarter had either a current procedure terminology (CPT) code or a P7 condition indicating admission through the ED, notably lower than in the non-LDC group (Table 1). Transfer-out patterns also differed among women who experienced LDCs compared to those who did not (Table 1). Patients with LDCs were less likely to remain at the index hospital (n= 1,006,760, 28.9% vs. n= 2,479,225, 71.1%) and more frequently required transfer, either to another acute care hospital (n= 1,810, 45.5%) or to another type of health facility (n= 930, 54.5%), compared with those without complications (Table 1). Pregnant women with renal calculi had a significantly longer mean hospital stay (5.12 ± 7.5 days) as compared to pregnant women without renal calculi (2.56 ± 2.2 days) (p < 0.001) (Table 1). 

**Table 1:** Comparison of patient and clinical characteristics by outcome group. Categorical variables are shown as counts (%), and continuous variables as mean ± SD. P-values (<0.001) indicate statistically significant differences between groups. HCUP: Healthcare Cost and Utilization Project; ED: emergency department; CPT: current procedure terminology; LDC: labor delivery complication

Characteristic (total n)	w/ LDC n (%)	w/o LDC n (%)	p-values
Renal Calculus (3,491,830)			<0.001
Present	6,510 (84.7)	1,180 (15.3)	
Absent	1,003,060 (28.8)	2,481,080 (71.2)	
HCUP ED Service Indicator (3,491,830)			<0.001
Record doesn’t meet any HCUP ED service indicator	836,140 (24.4)	2,107,475 (71.6)	
ED revenue code on record	125,570 (32.8)	257,380 (67.2)	
Positive ED charge w/revenue code unavailable	39,430 (29.8)	92,745 (70.2)	
ED CPT procedure code on record	955 (22.3)	3,320 (77.7)	
Condition code P7 indicates ED admission (originated or admitted)	7,475 (25.9)	21,340 (74.1)	
Patient Location (3,485,285)			<0.001
"Central" counties of metro areas (≥ 1 million population)	351,020 (31.8)	752,645 (68.2)	
"Fringe" counties of metro areas (≥ 1 million population)	256,245 (29.1)	624,949 (70.9)	
Counties in metro areas with 250,000 - 999,999 population	208,775 (28.2)	531,910 (71.8)	
Counties in metro areas with 50,000 - 249,999 population	77,990 (26.0)	221,775 (74.0)	
Micropolitan counties	68,640 (25.0)	206,086 (75.0)	
Not metropolitan or micropolitan counties	44,825 (24.2)	140,425 (75.8)	
Race (3,369,895)			<0.001
White	466,060 (27.1)	1,256,365 (72.9)	
Black	169,250 (34.7)	318,925 (65.3)	
Hispanic	228,720 (29.2)	555,435 (70.8)	
Asian or Pacific Islander	58,275 (29.3)	140,615 (70.7)	
Native American	8,260 (33.3)	16,565 (66.7)	
Other	46,300 (30.6)	105,125 (69.4)	
Transfer Out Indicator (3,491,665)			<0.001
Not a transfer	1,006,760 (28.9)	2,479,225 (71.1)	
Transferred out to a different acute care hospital	1,810 (45.5)	2,165 (54.5)	
Transferred out to another type of health facility	930 (54.5)	775 (45.5)	
Median Household Income National Quartile for Patient Zip Code (3,471,760)			<0.001
0 - 25th percentile	279,200 (29.8)	656,460 (70.2)	
26 - 50th percentile	245,540 (28.4)	617,780 (71.6)	
51 - 75th percentile	250,755 (28.7)	623,110 (71.3)	
76 - 100th percentile	227,915 (28.5)	571,000 (71.5)	
Primary Expected Payor (3,488,140)			<0.001
Medicare	7,390 (40.3)	10,955 (59.7)	
Medicaid	447,275 (31.1)	991,840 (68.9)	
Private insurance	503,775 (27.4)	1,337,565 (72.6)	
Self-pay	22,905 (25.9)	65,370 (74.1)	
No charge	840 (27.4)	2,225 (72.6)	
Other	26,375 (26.9)	71,625 (73.1)	
	Mean ± St. Dev.	Mean ± St. Dev.	
Gestational Age (GA)	37.8 ± 2.9	38.4 ± 2.0	<0.001
	w/ Renal Calculi Mean ± St. Dev.	w/o Renal Calculi Mean ± St. Dev.	
Length of Stay (LOS)	5.12 ± 7.5	2.6 ± 2.2	<0.001

**Figure 1:**
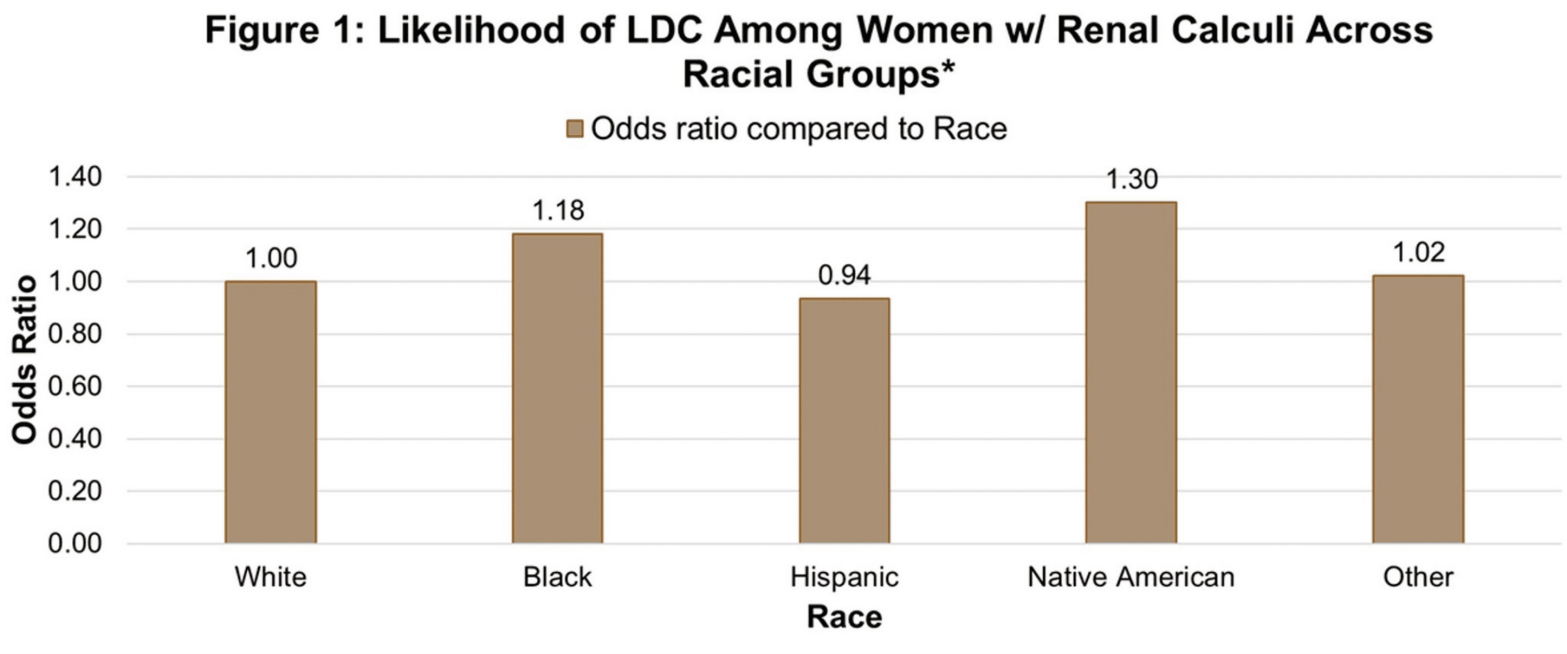
Likelihood of LDC among women w/renal calculi across racial groups. Adjusted odds of labor and delivery complications among women with renal calculi by race, with White women as the reference group (OR = 1.00). LDC: labor delivery complication

Insurance type was significantly associated with both renal calculi and delivery complications. Native American participants had the highest odds (AOR: 1.301) (95% CI: (1.265-1.338)) of LDC complications when compared to White participants (reference group), while Asian or Pacific Islander participants did not have a significantly increased risk of LDCs (Figure 1).

**Figure 2:**
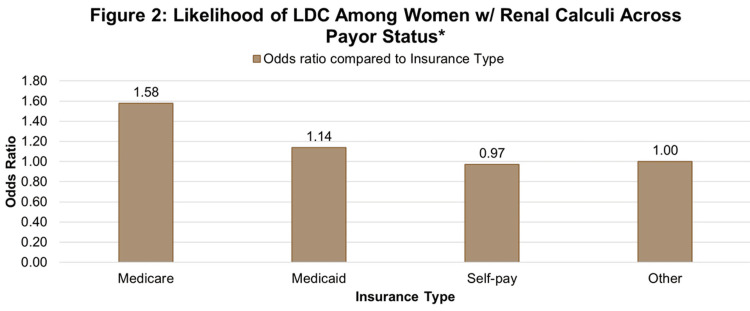
Likelihood of LDC among women w/renal calculi across payor status. Adjusted odds of labor and delivery complications among women with renal calculi by primary expected payor, with Other as the reference group (OR = 1.00). LDC: labor delivery complication

Compared to those with other insurance types, women with Medicare were more likely to experience a delivery complication and more likely to have renal calculi (aOR = 1.58) (95% CI: (1.523-1.633)), followed by those with Medicaid (aOR = 1.14) (95% CI: (1.120-1.155)), as shown in Figure 2.

**Figure 3:**
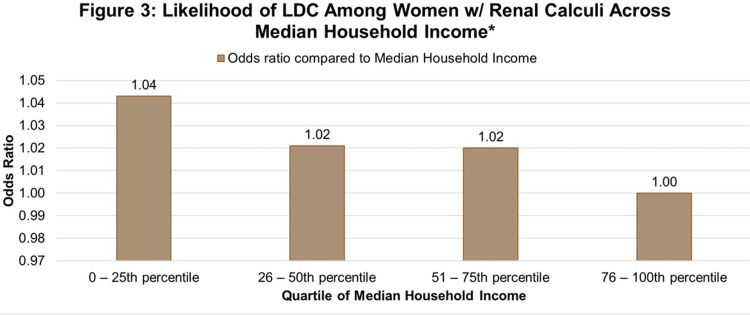
Likelihood of LDC among women w/renal calculi across median household income. Adjusted odds ratio of labor and delivery complications among women with renal calculi by Median Household Income National Quartile for patient ZIP Code, with the 76-100th percentile as the reference group (OR = 1.00). LDC: labor delivery complication

Private insurance holders (AOR: 1.012) (95% CI: (0.996-1.027)) were more likely to have higher complication rates compared to patients with other insurance (reference group) (Figure 2). Insurance type was significantly associated with both renal calculi and delivery complications. Women with lower annual incomes had significantly higher odds of LDCs compared to those from higher-income areas (Figure 3). 

## Discussion

This study demonstrated that renal calculi were a strong, independent predictor of LDCs in pregnant women. Insurance type was significantly associated with both renal calculi and LDCs. Compared to those with “other” insurance, those on Medicare had the highest adjusted odds for complications and renal calculi, followed by Medicaid (Figure 2). The increased risk of LDCs among Medicaid and Medicare patients aligns with prior research on insurance-related disparities in maternal mobility [16]. A prior study examined severe maternal morbidity and found Medicaid coverage was associated with increased risk of maternal morbidity (aOR 1.12) compared to privately insured deliveries [16]. The higher risk of LDCs among Medicaid and Medicare patients reflects insurance-related disparities and shows greater maternal morbidity with Medicaid coverage as compared to private insurance [16]. The observation that Medicare/Medicaid patients experience greater maternal morbidity compared with privately insured patients highlights a critical insurance-related disparity in obstetric outcomes. This is important because insurance status frequently serves as a proxy for socioeconomic vulnerability, limited access to consistent prenatal care, and reduced ability to obtain timely specialty evaluations [16]. From a health-systems standpoint, this disparity may signal structural inequalities within maternal healthcare delivery, leading to increased rates of LDCs in pregnant women with renal calculi. Standardizing management protocols for renal calculi in pregnancy may improve outcomes and reduce complications. 

Out of the patients with renal calculi, 84.7% experienced LDCs, whereas only 28.8% without renal calculi did (p < .001) (Table 1). These results are consistent with previous research findings, which report an increased risk of pre-eclampsia, LDC, urinary tract infection, low birth weight, and preterm labor in cases of renal calculi [1,17]. These findings underscore the substantial impact of renal calculi in pregnancy at a national level, demonstrating a higher resource utilization in pregnancies with LDCs and longer hospital stays among affected women [13].

Among patients with LDCs, only one-third had an ED revenue code, and one-quarter had indicators of ED admission, suggesting many were admitted through alternative routes such as direct admission or interfacility transfer. Compared to the non-LDC group, this pattern may reflect differences in acuity, access to emergency care, or institutional practices [10]. Additionally, higher transfer-out rates in the LDC group may indicate greater clinical complexity or limited resources, warranting further investigation into care pathways and systemic disparities in obstetric management [13]. The increased rates of transfer-out patterns among patients with LDCs as indicated in Table 1 are consistent with expectations for pregnant women with renal calculi, who are often referred to tertiary centers for definitive care (e.g., urologic intervention) [18]. It was found that pregnant women from rural areas were more likely to experience an LDC as compared with those from urban settings, which is consistent with national data that rural mothers experience greater rates of maternal mortality than those in metropolitan areas. This is often due to reduced access to prenatal and specialty care, longer travel times, and shortages of obstetric providers [12,19,20]. These patterns may reflect differences in patient risk profiles, referral and delivery practices, or underdiagnosis of complications in rural settings. Future research may lead to improvements in obstetric resources in rural hospitals, in an effort to reduce inequities in maternal outcomes. 

Prior research has found that pregnant patients with renal calculi most often present through the ED for initial evaluation and management [10]. In contrast, Table 1 shows lower ED service indicators captured in pregnant women with LDCs. This finding likely reflects coding limitations and direct admission through labor and delivery triage rather than a true difference in care pathways [15].

A key limitation of this study is that ICD-10 codes do not distinguish renal caliculi types. Grouping all renal calculi together likely underestimates the impact of more severe forms, such as staghorn calculi, which carry higher risks, require more intensive treatment, and present unique management challenges during pregnancy [21]. The HCUP-NIS database also precludes the ability to determine whether LDCs were directly attributable to renal calculi or to comorbid conditions. Considering that HCUP-NIS is a hospital discharge database, readmissions are unable to be linked to individual patients, and patients who are seen in the emergency room, treated, and then discharged are also not included in the dataset. This prevents the assessment of longitudinal outcomes such as recurrence, postpartum stone events, or long-term maternal and neonatal complications. However, a strength of using HCUP-NIS is its large, nationally representative sample drawn from the largest publicly available all-payer inpatient database in the United States, which enhances the generalizability of findings to pregnant women across diverse hospital settings. Few large-scale studies have examined the relationship between renal calculi and LDC. This analysis addresses a gap in the literature by quantifying risks at a national level. 

In future studies, limitations could be further evaluated and addressed. Distinguishing between the different types of renal calculi (staghorn, calcium oxalate, struvite, cysteine, and uric acid) within the data would help to determine complications each stone may induce, or if the stone type contributes to increased LDC and/or maternal-fetal complications. Longitudinal studies, such as prospective cohort studies, could be considered to track long-term outcomes, assess postpartum outcomes, track renal calculi recurrence, and assess neonatal complications. Lastly, rural-urban findings could be further investigated to distinguish true epidemiological trends and disparities in diagnosis or access to care. Evaluating these gaps in future research could clarify risk patterns and improve targeted management of renal calculi in pregnancy. 

## Conclusions

Renal calculi are strongly associated with adverse maternal outcomes, with a disproportional burden among those with Medicaid and Medicare coverage and patients who reside in rural areas. The findings underscore the need for standardized management protocols and targeted interventions to address insurance-related and geographic disparities in maternal care. By highlighting underrecognized patterns in admission pathways and transfer rates, this study identifies overlooked care patterns that may inform maternal risk stratification and support more equitable, evidence-based management of renal calculi in pregnancy. Future research should focus on risks specific to renal calculi type and optimal management strategies during pregnancy to improve maternal and neonatal outcomes.
